# Distribution and dynamics of epidemic and pandemic *Vibrio parahaemolyticus* virulence factors

**DOI:** 10.3389/fcimb.2013.00097

**Published:** 2013-12-11

**Authors:** Daniela Ceccarelli, Nur A. Hasan, Anwar Huq, Rita R. Colwell

**Affiliations:** ^1^Maryland Pathogen Research Institute, University of MarylandCollege Park, MD, USA; ^2^CosmosID Inc.College Park, MD, USA; ^3^Maryland Institute of Applied Environmental Health, University of MarylandCollege Park, MD, USA; ^4^Department of Environmental Health Sciences, Johns Hopkins Bloomberg School of Public Health, Johns Hopkins UniversityBaltimore, MD, USA

**Keywords:** *V. parahaemolyticus*, O3:K6, *tdh*, *trh*, PAI, virulence markers, pandemic strains, epidemic strains

## Abstract

*Vibrio parahaemolyticus*, autochthonous to estuarine, marine, and coastal environments throughout the world, is the causative agent of food-borne gastroenteritis. More than 80 serotypes have been described worldwide, based on antigenic properties of the somatic (O) and capsular (K) antigens. Serovar O3:K6 emerged in India in 1996 and subsequently was isolated worldwide, leading to the conclusion that the first *V. parahaemolyticus* pandemic had taken place. Most strains of *V. parahaemolyticus* isolated from the environment or seafood, in contrast to clinical strains, do not produce a thermostable direct hemolysin (TDH) and/or a TDH-related hemolysin (TRH). Type 3 secretion systems (T3SSs), needle-like apparatuses able to deliver bacterial effectors into host cytoplasm, were identified as triggering cytotoxicity and enterotoxicity. Type 6 secretion systems (T6SS) predicted to be involved in intracellular trafficking and vesicular transport appear to play a role in *V. parahaemolyticus* virulence. Recent advances in *V. parahaemolyticus* genomics identified several pathogenicity islands (VpaIs) located on either chromosome in both epidemic and pandemic strains and comprising additional colonization factors, such as restriction-modification complexes, chemotaxis proteins, classical bacterial surface virulence factors, and putative colicins. Furthermore, studies indicate strains lacking toxins and genomic regions associated with pathogenicity may also be pathogenic, suggesting other important virulence factors remain to be identified. The unique repertoire of virulence factors identified to date, their occurrence and distribution in both epidemic and pandemic strains worldwide are described, with the aim of highlighting the complexity of *V. parahaemolyticus* pathogenicity as well as its dynamic genome.

## Introduction

Some pathogenic bacteria occur naturally in coastal waters worldwide, of which a few can cause disease outbreaks, depending on specific environmental conditions. A good example is *Vibrio parahaemolyticus*, a Gram-negative halophilic bacterium autochthonous to estuarine, marine, and coastal environments. Occupying a variety of niches, *V. parahaemolyticus* can exist in a free-swimming state, with its motility conferred by a single polar flagellum, or sessile, attached to inert and animate surfaces, such as suspended particulate matter, zooplankton, fish, and shellfish (McCarter, 1999). Depending on environmental conditions, *V. parahaemolyticus* can produce a capsule with a number of different somatic (O) and capsular (K) antigens and these are employed as a primary basis of strain classification (Nair et al., 2007). Distribution of *V. parahaemolyticus* in the marine environment is related to water temperature and it is rarely isolated from seawater until the temperature rises to 15°C and higher (Kaneko and Colwell, 1973). Contamination of raw shellfish by *V. parahaemolyticus* is also known to be related to water temperature and it is more likely to occur during spring and summer months.

Since its discovery in 1950 (Fujino et al., 1953), *V. parahaemolyticus* has been recognized as a leading cause of seafood-derived food poisoning throughout the world. Virulent strains transmitted via consumption of raw or undercooked seafood are a common cause of acute gastroenteritis (Newton et al., 2012). Although the gastroenteritis may be self-limited, the infection can cause septicemia that is life-threatening to those with a preexisting medical conditions (Su and Liu, 2007). In Japan, *V. parahaemolyticus* is responsible for 20–30% of all food poisoning cases (Alam et al., 2002) and is the common cause of seafood-borne illness in many Asian countries (Koralage et al., 2012; Yu et al., 2013). *V. parahaemolyticus* has also become the leading agent of human gastroenteritis associated with seafood consumption in the United States (Newton et al., 2012).

The global occurrence of *V. parahaemolyticus* emphasizes the importance of understanding its many virulence factors and their effect on the human host. The objective of this study, therefore, was to review *V. parahaemolyticus* virulence associated factors that have been identified and described to date, and employ this information to provide a better understanding of *V. parahaemolyticus* pathogenesis.

## *V. parahaemolyticus* distinctive virulence factors, *tdh* and *trh*

Although *V. parahaemolyticus* is a natural inhabitant of the estuarine and marine environment, only some strains have proven to be pathogenic (Oliver and Kaper, 2007). Almost all environmental isolates are negative in experimental animal diarrhogenic tests, whereas isolates from patients are positive (Shinoda, 2011). To date, the most distinctive factors of virulent strains are the thermostable direct hemolysin (TDH) (Nishibuchi et al., 1992) and TDH-related hemolysin (TRH) (Honda et al., 1988). Almost all *V. parahaemolyticus* strains isolated from clinical samples possess beta-hemolytic activity attributed to these two genes. Such isolates are able to lyse human erythrocytes when plated on high-salt Wagatsuma agar, a process designated as the Kanagawa phenomenon (KP) (Miyamoto et al., 1969; Joseph et al., 1982).

TDH is an amyloid toxin with two potential activities, one of which is disruption of lipid microdomains, abrogating cytotoxicity in the cell (Matsuda et al., 2010). The fairly large pore size allows both water and ions to flow through the membrane (Honda et al., 1992). These alterations in ion flux in the intestine ultimately prove to be the mechanism of the diarrhea observed during infection. However, even in the absence of these thermostable hemolysins *V. parahaemolyticus* remains pathogenic, indicating other virulence traits exist (Nishibuchi et al., 1992; Xu et al., 1994). Several *tdh* alleles sharing high nucleotide homology (>97%) have been identified on both chromosomes and plasmids (Nishibuchi and Kaper, 1995). The intensity of the hemolysis has also been associated with different transcriptional control and *tdh* gene copy in strains of *V. parahaemolyticus* (Nishibuchi and Kaper, 1990). The *tdh* gene is absent in most environmental strains of *V. parahaemolyticus*, but present in some strains of *V. mimicus*, *V. cholerae* non-O1/non-O139 and *V. hollisae* (Nishibuchi and Kaper, 1995; Shinoda, 2011).

KP-negative clinical strains of *V. parahaemolyticus* not possessing the *tdh* gene have been shown to produce a second hemolysin, TRH, which, unlike TDH, is heat labile and immunologically similar to TDH (Honda et al., 1988). EB101, the type strain of *V. parahaemolyticus* (Fujino et al., 1953) was reported to be KP-negative and later identified as *tdh*^−^/*trh*^+^ by molecular biological analysis (Shinoda, 2011). Both hemolysins (*trh* and *tdh)* share approximately 70% homology, with *trh* having higher nucleotide variability among alleles (Kishishita et al., 1992). TRH, believed to act similarly to TDH by activating Cl^−^ channels, resulting in altered ion flux (Takahashi et al., 2000), was recently identified in *Aeromonas veronii* isolates (Raghunath et al., 2010).

Although correlation between pathogenicity of *V. parahaemolyticus* and presence of *tdh* and *trh* is well accepted, it should also be noted that not all clinical strains possess these genes. Several studies have reported about 10% of clinical strains do not contain *tdh* and *trh*, with the result that the overall mechanism of *V. parahaemolyticus* pathogenesis remains unclear (Miyamoto et al., 1969; Shirai et al., 1990; Okuda et al., 1997a). Association of a predominant serotype with human disease, namely *V. parahaemolyticus* O3:K6, and achievement of the first complete genome sequencing have shed some light on the virulence and epidemiological characteristics of *V. parahaemolyticus*.

## *V. parahaemolyticus* O3:K6 as landmark of epidemiology

At the beginning of 1996 in Kolkata, India, during ongoing surveillance, an increase in patients with *V. parahaemolyticus* gastroenteritis was observed. Analysis of the strains revealed a new unique serotype, O3:K6, which accounted for 50–80% of infections during the following months. All O3:K6 isolates were *tdh*^+^/*trh*^−^ and shared nearly identical genotype profiles (Okuda et al., 1997b). Within a few months thereafter, strains of the same serogroup were isolated in the neighboring countries of Vietnam, Indonesia, Bangladesh, Laos, Japan, Korea, and Thailand (Nair et al., 2007). Specific methods to identify the new clone, based on variation in nucleotide sequence of the *toxRS* region, were employed (Matsumoto et al., 2000) and the results showed existence of strains almost indistinguishable from the “new” O3:K6 clone, even though the isolates belonged to different serovars (Chowdhury et al., 2000a). To date, 21 serotypes have been identified, collectively referred to as serovariants of O3:K6, the most common being O4:K68, O1:K25, O1:K41, and O1:KUT (Nair et al., 2007). As anticipated by Chowdhury and colleagues, these serotypes differ from O3:K6 in altered O:K antigens, but are of clonal origin (Chowdhury et al., 2000a). By the end of 2006, *V. parahaemolyticus* O3:K6 and its serovariants were being isolated in Europe, Mozambique, the United States, Mexico, and South American countries (Nair et al., 2007), marking what has been claimed to be the beginning of the first pandemic of *V. parahaemolyticus*, bringing this pathogen to the forefront of the global public health agenda.

It is not obvious what environmental factors were associated with the global reach of these pandemic strains. Several efforts were made to determine factor(s) giving *V. parahaemolyticus* O3:K6 the ability to cause an increase in hospitalizations and to become the dominant serotype. Increased production of TDH was rapidly disproven since no substantial difference was detected between pandemic and non-pandemic strains (Okuda et al., 1997b). The *V. parahaemolyticus* O3:K6 and earlier isolates did not differ in survival under the same environmental conditions, such as extreme temperature, low pH, and/or elevated salinity (Wong et al., 2000). Yeung and colleagues suggested enhanced adherence and cytotoxicity may contribute to the pathogenic potential of *V. parahaemolyticus* O3:K6 isolates (Yeung et al., 2002), but the real advantage of O3:K6 over other strains remains unclear. Strains belonging to pandemic O3:K6 or related serogroups have been isolated from environmental samples in several countries, including Bangladesh (Islam et al., 2004), Japan (Hara-Kudo et al., 2003), India (Deepanjali et al., 2005), and Italy (Caburlotto et al., 2010a) suggesting they may prove endemic, if favorable niches occur in the environment.

## Pandemic vs. non-pandemic strains and virulence characteristics

What has clearly emerged from analysis of *V. parahaemolyticus* O3:K6 strains and comparison with other serotypes is that the genetic organization of *V. parahaemolyticus* is more complex than expected. Results of molecular analysis, including ribotyping, AP-PCR, and PFGE (Okuda et al., 1997b; Chowdhury et al., 2000a,b; Matsumoto et al., 2000) showed O3:K6 isolates are genetically similar to each other and distinct from the O3:K6 strains isolated before 1995 and non-O3:K6 strains. Post-1995 *V. parahaemolyticus* O3:K6 isolates carry *tdh* but not *trh* and are defined by pandemic group-specific PCR (PGS-PCR) (Okura et al., 2003, 2004) and by a unique/new *ToxR* sequence within the *toxRS* operon, showing a slightly different nucleotide sequence compared to non-pandemic strains and likely involved in regulation of conserved virulence-associated genes in species of the genus *Vibrio* (Matsumoto et al., 2000).

These initial findings were the basis of the definition of pre- and post-pandemic *V. parahaemolyticus* strains of other serogroups beside O3:K6 (or serovariants) isolated before and after 1995. Differences observed among and between O3:K6 strains led to the definition of non-pandemic O3:K6 (or serovariants) as strains isolated between 1980–1990 in several Asian countries, including India, Taiwan, Japan, Thailand, and Bangladesh (Okuda et al., 1997b; Matsumoto et al., 2000; Osawa et al., 2002; Wong et al., 2005).

Nasu and colleagues reported that a wide collection of O3:K6 isolates had in common the filamentous phage f237 and suggested a specific association between the phage and widespread O3:K6 serotype (Nasu et al., 2000). Furthermore, they suggested that ORF8, located in the phage and encoding a putative adherence protein specific to post-1995 *V. parahaemolyticus* O3:K6 strains, might have played a significant role in increasing the virulence of O3:K6 isolates by their being more adhesive to host intestinal cells. However, many post-1995 *V. parahaemolyticus* strains lack phage f237 (Chowdhury et al., 2000b; Bhuiyan et al., 2002). The possible role of phages in pandemic *V. parahaemolyticus* infection was also suggested by identification of VfO4K68 in *V. parahaemolyticus* O4:K68 strains (Chan et al., 2002).

Okura and colleagues discovered that pandemic strains share the gene sequence VP2905, while non-pandemic group strains do not (Okura et al., 2005). This gene is located in a 16-kb region inserted in the open reading frame of the histone-like DNA-binding protein HU-α, causing a frameshift in the amino acid sequence (Williams et al., 2004). In enteric bacteria, this class of proteins has been associated with modification in drug resistance (Nishino and Yamaguchi, 2004). In *Streptococcus* it is related to tissue inflammation (Stinson et al., 1998). The effect on *V. parahaemolyticus* pathogenicity is unknown at this time.

Although these genetic traits appeared to be suitable markers for identification of pandemic strains, inconsistencies have been noted whereby pandemic O3:K6 strains with atypical profiles (lack of *tdh* or f237) have been isolated in Taiwan, Bangladesh, Japan, and Thailand (Bhuiyan et al., 2002; Osawa et al., 2002; Chowdhury et al., 2004; Jones et al., 2012) highlighting the complexity of pandemic *V. parahaemolyticus*, leaving genotyping an open question.

## The first sequenced *V. parahaemolyticus* genome and discovery of T3SS

Pandemic *V. parahaemolyticus* O3:K6 strain RIMD 2210633 was isolated in Japan at the Kansai International Airport quarantine station in 1996 from a patient with travelers' diarrhea (Nasu et al., 2000). Makino and colleagues described its genome as comprising two circular chromosomes, with 4832 genes and showing rearrangements within and between the two chromosomes and the *V. cholerae* genome (Makino et al., 2003). A new type III secretion system, T3SS1, located on chromosome 1 was identified. Type 3 secretion systems (T3SSs) are a needle-like bacterial machine used to inject bacterial effectors directly into the membrane and cytoplasm of eukaryotic cells without encountering the extracellular environment (Cornelis, 2006). The T3SS1 gene cluster is composed of 42 genes, of which 30 are similar to those of other T3SS, e.g., *Pseudomonas aeruginosa* and *Yersinia* sp. (Makino et al., 2003; Park et al., 2004a). Three main effectors from T3SS1 were described: (i) VopQ induces rapid induction of autophagy in target cells preventing phagocytosis of the infecting bacteria (Sreelatha et al., 2013); (ii) VPA0450 destabilizes the cell by detachment of the plasma membrane from the actin cytoskeleton (Broberg et al., 2010); and (iii) VopS promotes collapse of the actin cytoskeleton, leading to cell rounding and shrinkage (Yarbrough et al., 2009). The role of a fourth effector located within the T3SS1 gene locus, VopR, has not been determined (Broberg et al., 2011). Collectively, T3SS1 effectors help *V. parahaemolyticus* to evade the host immune response, inducing autophagy followed by cell rounding and cell lysis (Higa et al., 2013). T3SS1 activities appear to be highly regulated by a dual system similar to the ExsACDE regulatory cascade of *P. aeruginosa* and negative regulation by H-NS (Kodama et al., 2010). Sharing a certain degree of homology with *Yersinia* spp. and other *Vibrio* species systems, the T3SS1 high sequence homology suggests these genes were ancestrally acquired and have been evolutionarily conserved (Ham and Orth, 2012). T3SS1 is well conserved and widespread in both clinical and environmental strains of *V. parahaemolyticus* (Park et al., 2004a; Meador et al., 2007; Noriea et al., 2010), being an essential characteristic to this species.

In addition to T3SS1, Makino et al. (2003) identified a pathogenicity island (Vp-PAI, currently referred to as VPaI-7) on chromosome 2 encoding several virulence-related genes, such as homologs of the *Escherichia coli* cytotoxic necrotizing factor (CNF) and *Pseudomonas* exoenzyme T (Makino et al., 2003). Associated with VPaI-7, typically flanked by two *tdh* genes, is a second type III secretion system, T3SS2-α, not similar to any T3SS of other bacteria, suggesting it is intrinsic to the species. Recent findings demonstrated that, along with its six effectors, T3SS2-α allows *V. parahaemolyticus* to invade, survive, and replicate in non-phagocytic host cells (Zhang et al., 2012). VopC has homologies to CNF1 described in pathogenic *E. coli*, and activates small GTPases Rac and CDC42 to induce changes in the actin cytoskeleton and facilitate *V. parahaemolyticus* entry into nonphagocytic host cells (Zhang et al., 2012). VopT is an ADP-ribosyltransferase able to modify the small GTPase Ras (Kodama et al., 2007) and is likely to have many effects during host infection such as cytotoxicity. VopA/P blocks activation of the MAPK signaling pathway which prevents induction of cytokines (Trosky et al., 2004). VopL polymerization activity is responsible for the strong actin filament nucleation observed in the host cell (Namgoong et al., 2011). VopV encodes an F-actin-binding protein involved in *V. parahaemolyticus* enterotoxicity (Hiyoshi et al., 2011). Finally, recently identified VopZ inhibits kinase TAK1 activation and is essential for *V. parahaemolyticus* induced diarrhea and tissue disruption (Zhou et al., 2013).

The co-presence of *tdh* genes and T3SS2-α on Vp-PAI was investigated and *tdh* was found to be co-regulated with T3SS2-α genes (Park et al., 2004a,b; Gotoh et al., 2010), suggesting a specific role of T3SS2-α and *tdh* in *V. parahaemolyticus* pathogenicity. Interestingly, T3SS2 was found only in Kanagawa-phenomenon-positive isolates and associated with enterotoxicity, whereas T3SS1 is ubiquitous in *V. parahaemolyticus* strains and correlated with cytotoxic activity (Park et al., 2004a,b; Meador et al., 2007; Izutsu et al., 2008).

Following the observation that *trh* could be located on a pathogenicity island (Iida et al., 1998), Okada and colleagues analyzed the surrounding region of the *trh* gene in strain TH3996 and discovered a novel T3SS2-α homolog inserted in pathogenicity island Vp-PAI_TH3996_ (a.k.a. *tdh*PAI), T3SS2-β (Okada et al., 2009; Chen et al., 2011). They concluded that the new T3SS-related gene cluster does not occur in KP-positive strains, indicating a distinct lineage of T3SS2-related genes in *tdh*^−^ and *trh*^+^ *V. parahaemolyticus* strains.

It was believed T3SS2-α and *tdh* were distinctive features of the TDH-producing (Kanagawa-phenomenon-positive) strains, exclusive to clinical isolates and associated with pandemic *V. parahaemolyticus* strains (Baker-Austin et al., 2010; Broberg et al., 2011). As in *tdh*^+^ strains, *trh* is linked with T3SS2-β, identified only in *trh*^+^ *V. parahaemolyticus* strains (Ottaviani et al., 2012). Recent findings question a direct correlation between T3SSs and *tdh*/*trh* genes. T3SS2-α and T3SS2-β, respectively, were found in *tdh*^+^/*trh*^−^and*tdh*^−^/*trh*^+^ strains, as expected, but also in *V. parahaemolyticus* environmental and clinical isolates, for which presence of *tdh* and *trh* was variable (Jones et al., 2012; Paranjpye et al., 2012).

Understanding the relationship between *tdh*, *trh*, and type III secretion systems in environmental isolates is of vital importance for validating hemolysin genes as virulence markers, as well as understanding the pathogenic potential of environmental strains.

## Comparative genomics: pathogenicity islands and the dynamic genome of *V. parahaemolyticus*

Unlike *V. cholerae*, for which the genomes of well over 100 strains have been sequenced over the past 13 years, only a few *V. parahaemolyticus* strains have been completely sequenced and/or annotated to date (Table 1) (Boyd et al., 2008; Chen et al., 2011; Gonzalez-Escalona et al., 2011; Jensen et al., 2013; Jun et al., 2013; Liu and Chen, 2013). Nevertheless, since the first strain, RIMD 2210633, was sequenced (Makino et al., 2003), several comparative studies have identified new genomic islands (GIs). Acquisition of new genetic material by horizontal transfer plays a pivotal role in shaping the *V. parahaemolyticus* genome.

**Table 1:** ***V. parahaemolyticus* strains that have been sequenced**.

**Strain**	**Isolation (date and location)**	**Serotype**	***tdh/trh***	**T3SSs/T6SS**	**Origin**	**Sequencing status**	**References**
**PRE-PANDEMIC**							
BB22OP	1980s, Bangladesh	O4:K8	+/−	+/+	Env	Complete	Jensen et al., 2013
AQ3810	1983, Singapore	O3:K6	+/−	+/−	Clin	Draft	Boyd et al., 2008
AQ4037	1985, Maldive	O3:K6	−/+	+/+	Clin	Draft	Chen et al., 2011
**PANDEMIC**							
RIMD 2210633	1996, Japan	O3:K6	+/−	+/+	Clin	Complete	Makino et al., 2003
Peru466	1996, Peru	O3:K6	+/−	+/+	Clin	Draft	Chen et al., 2011
K5030	2005, India	O3:K6	+/−	+/+	Clin	Draft	Chen et al., 2011
**NON-PANDEMIC**							
10329	1998, USA	O4:K12	+/+	ND/ND	Clin	Draft	Gonzalez-Escalona et al., 2011
AN5034	1998, Bangladesh	O4:K68	+/−	+/+	Clin	Draft	Chen et al., 2011
SNUVpS-1	2009, Korea	ND	−/−	ND/ND	Env	Draft	Jun et al., 2013
v110	2010, Hong Kong	ND	−/−	+^*^/ND	Env	Draft	Liu and Chen, 2013

Env, environmental; Clin, clinical; ND, not determined.

* Only T3SS1.

GIs are chromosomal regions usually acquired by horizontal gene transfer. They carry genes that can confer fitness advantage to their bacterial host. GIs encoding virulence determinants or colonization factors promote pathogenicity or survival of the bacterium in the host and are referred to as pathogenicity islands (Dobrindt et al., 2004).

Today it is known that *V. parahaemolyticus* contains nine GIs, VPaI-1 to VPaI-9, located on both chromosomes and variably distributed among different isolates (Hurley et al., 2006; Boyd et al., 2008). The nine GIs possess a phage-like integrase gene and are flanked by direct repeat sequences, with the exception of VPaI-7 that lacks an integrase but contains several transposase genes (Hurley et al., 2006). GIs range in size between 10 and 81 kb and their G+C content is lower than the overall genome G+C content (~45%) suggesting that these regions were acquired by horizontal gene transfer (Hurley et al., 2006; Boyd et al., 2008). The possible origin of the VPaIs is still not clearly understood. Blast analysis revealed the presence of homolog ORFs to some of the proteins encoded in VPaI-1, VPaI-2, VPaI-3, and VPaI-5 in different strains of *V. cholerae*, *V. harveyi*, and *Shewanella* sp. (Boyd et al., 2008). VPaIs have no close homologs in other *Vibrionaceae* but share the same insertion sites into tRNA-Met, tRNA-Ser or tmRNA loci as key pathogenicity islands of *V. cholerae* O1 (VPI-1, VPI-2, and VSP-2) and *V. vulnificus* (VVI-1, VVI-3, and VVI-7) (Hurley et al., 2006).

Analysis of VPaI-1 revealed a 22.79 kb pathogenicity island containing 24 open reading frames encoding proteins involved in DNA replication, transcription regulation, signal transduction, and general metabolism, as well as a type I restriction-modification complex and a DNA methyltransferase gene (Wang et al., 2006). By analogy with the mannose-fucose-resistant hemagglutinin (MFRHA) of *V. cholerae* O1 (Franzon et al., 1993), it is assumed that the DNA methyltransferase VP0394 comprises an additional colonization factor. Interestingly, the presence of this gene is strongly associated with *tdh*^+^ pandemic isolates, suggesting that VP0394 may confer unique virulence or fitness traits to these strains (Hurley et al., 2006; Wang et al., 2006). Comparison of VPaI-1 with other bacterial genomes revealed that an 8 kb region containing genes VP0389, VP0390, VP0391, and VP0392 is syntenic with chromosomal regions found in *V. vulnificus* CMCP6 (VVI-2) and *Shewanella* sp. MR-7 (Nishioka et al., 2008). The possible role of VP0390 and VP0392 in the cold adaptation of post-1995 *V. parahaemolyticus* strains has been hypothesized but further work needs to be done.

VPaI-2 to VPaI-6 range in size between 10 and 32 kb and encode putative virulence genes potentially involved in *V. parahaemolyticus* pathogenicity: outer membrane proteins and resolvases (VPaI-2); methyl accepting chemotaxis proteins (VPaI-3); putative pore forming cytotoxin integrase and M proteins likely involved in classical bacterial surface virulence factors (VPaI-4); phage-like protein that may encode a phage (VPaI-5); and putative colicin proteins (VPaI-6) (Hurley et al., 2006).

Involvement of VPaI-7 (formerly known as Vp-PAI, 8 kb) in cytotoxicity and enterotoxicity of *V. parahaemolyticus* has been described and correlated with presence of *tdh* and T3SS2-α (Makino et al., 2003; Park et al., 2004a,b; Sugiyama et al., 2008). Microarray analysis demonstrated association of VpaI-7 with pandemic strains (Izutsu et al., 2008). Comparison of *V. parahaemolyticus* O3:K6 and O4:K68 pandemic strains showed their genomes are similar in content. Furthermore, comparison between KP-positive and KP-negative strains highlighted that only the former contain VPaI-7, strongly suggesting that not only *tdh* but the entire region is required for pathogenicity of KP-positive clinical *V. parahaemolyticus* strains (Izutsu et al., 2008). Absence of VPaI-7 in KP-negative strains is compensated by Vp-PAI_TH3996_ typical of *tdh*^−^/*trh*^+^ strains, encoding T3SS2-β (Okada et al., 2009). Recent studies suggest that, similar to clinical strains, marine isolates of *V. parahaemolyticus* carrying the T3SS2 effectors VopT and VopB2 and other genes included in VPaI-7 are capable of adhering to human cells and also causing cytoskeletal disruption and loss of membrane integrity in infected cells (Caburlotto et al., 2010b). These findings support the consideration of environmental *V. parahaemolyticus* strains as a risk to human health.

GIs VPaI-8 and VPaI-9 have recently been described and appear to be a feature of *tdh*^+^ pre-pandemic strains, namely AQ3810 (Boyd et al., 2008). VPaI-8 is 17 kb long and contains several ORFs encoding hypothetical proteins, two integrases, and homologs of KAP proteins. The latter are implicated in nearly all biochemical and mechanical processes in the cell, including replication and repair, intracellular trafficking, membrane transport, and activation of various metabolites (Aravind et al., 2004). VPaI-9 is a 22 kb region and, among other functions, encodes an excisionase, an helicase and a type I restriction modification system (Boyd et al., 2008). The role of these two GIs in *V. parahaemolyticus* pathogenicity has not been determined.

During the past few years GIs have been used as genetic markers to identify pandemic strains, with VPaI-1, VPaI-4, VPaI-5, and VPaI-6 found to be unique to post-1995 pandemic *V. parahaemolyticus* (Hurley et al., 2006), as well as VPaI-3 (Izutsu et al., 2008). VPaI-2 was described, not only in isolates post-1995 but also in isolates before 1995, prior to acquisition by the pandemic strains (Boyd et al., 2008).

Unsurprisingly, the scenario is more complex, as established by several exceptions. Not all pandemic strains contain VPaI-1 and VPaI-5 and *tdh*^+^ non-pandemic strains can be associated with VPaI-7 (Chao et al., 2009). Pandemic strains devoid of VPaI-4 have also been described, whereas VPaI-6 is common in non-pathogenic, pathogenic, and pandemic strains, indicating non-uniqueness of VPaI-6 to the pandemic clone (Chao et al., 2010). The presence of genomic regions characteristic of the pandemic clone in other non-pandemic strains provides evidence of genetic transfer, undoubtedly a major force in shaping virulence of *V. parahaemolyticus*.

## T6SS, the ultimate weapon to compete for new niches

Comparison between pandemic and non-pandemic strains of *V. parahaemolyticus* led to identification of type VI secretion systems, T6SS1 and T6SS2, located on chromosome 1 and 2 of *V. parahaemolyticus* RIMD 2210633, respectively (Boyd et al., 2008; Izutsu et al., 2008). Homologs of Type 6 secretion system (T6SS) are present in *V. alginolyticus*, *V. harveyi*, and *V. cholerae* and are predicted to be involved in intracellular trafficking and vesicular transport (Boyd et al., 2008).

The role of T6SS2 is under analysis, but preliminary data suggest that it is not involved in cytotoxicity, as is the case for other bacterial T6SSs (Pukatzki et al., 2007), but is functional in adhesion to host cells (Yu et al., 2012). Functionality of T6SS1 has not yet been demonstrated. Since T6SS2 and T3SS2 co-exist, it was proposed that the two systems might cooperate during infection. T6SS2 plays its role in adhesion, the first step of infection, and T3SS2 exports effectors by inducing enterocytotoxicity (Park et al., 2004a; Yu et al., 2012).

A role for T6SSs in environmental fitness of *V. parahaemolyticus* has also been proposed. Salomon and colleagues elegantly established that T6SS1 is most active under warm marine-like conditions, while T6SS2 is active under low salt conditions and that surface sensing and quorum sensing differentially regulate both systems (Salomon et al., 2013). Moreover, they confirmed that *V. parahaemolyticus* employs T6SS1 to compete against other bacterial species, as well as against strains of its own species. This represents an advantage during summer months, when coastal waters are warm and increase in marine bacterial populations requires *V*. *parahaemolyticus* to compete for a niche (Salomon et al., 2013).

*V. parahaemolyticus* T6SS was used as a virulence marker to differentiate strains. Chao and colleagues reported that most pandemic strains isolated in China had the complete set of T6SS genes, whereas the majority of non-pathogenic strains had a partial set of T6SS genes (Chao et al., 2010). These results are consistent with the hypothesis that the entire set of T6SS genes is associated with pandemic strains (Boyd et al., 2008).

## Conclusion and future perspectives

It is evident that the genome of *V. parahaemolyticus* is highly versatile, and the presence of pandemic genomic regions in non-pandemic strains provides evidence for horizontal gene transfer, shaping virulence, and evolution of *V. parahaemolyticus*.

**Figure 1:**
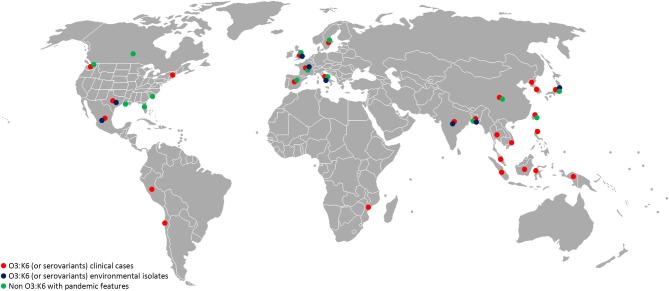
**Worldwide distribution of *V. parahaemolyticus* featuring virulence markers**. Red, O3:K6 (or serovariants) clinical isolates; blue, O3:K6 (or serovariants) environmental isolates (water, seafood); green, non O3:K6 strains showing pandemic features (*tdh*, *trh*, PAI, T3SS, and/or T6SS). References to these data are a small portion of the published studies and should guide as an example. (Myers et al., 2003; Islam et al., 2004; Quilici et al., 2005; Nair et al., 2007; Ellingsen et al., 2008; Ottaviani et al., 2008, 2010b; Chao et al., 2009, 2010; Baker-Austin et al., 2010; Jones et al., 2012; Velazquez-Roman et al., 2012; Powell et al., 2013).

Virulence genes and pathogenicity islands are present in environmental strains and acquisition of these genes very likely occurs in the aquatic environment. Non-pathogenic *Vibrio* strains isolated in the Venetian Lagoon were found to contain remnants of *V. parahaemolyticus* VPaI-7, along with *V. cholerae* neuraminidase *nanH* and a modified version of pathogenicity island VPI-2 (Gennari et al., 2012). Also, environmental strains of non-O3:K6 *V. parahaemolyticus* carrying genetic markers associated with pandemic strains (*tdh*, *orf8*, *toxRS*/new) were recently isolated in Europe, the United States, Mexico, and Bangladesh (Alam et al., 2009; Caburlotto et al., 2010a; Jones et al., 2012; Velazquez-Roman et al., 2012) (Figure 1). These findings support the view that estuarine and marine bacteria comprise a significant reservoir of virulence and fitness genes. Such genes may provide selective advantage in the aquatic environment, enabling the bacteria to persist in the environment, with greater chance of encountering a susceptible host or exchange genetic material with other members of marine microbial communities in the same ecological niche. Exchange of genetic material occurs among autochthonous estuarine and marine bacteria and also human pathogens released via anthropogenic activities to the aquatic environment. Coastal water contamination with *V. parahaemolyticus* O3:K6 has been reported worldwide (Myers et al., 2003; Islam et al., 2004; Quilici et al., 2005; Ottaviani et al., 2010a; Powell et al., 2013). Clearly pathogenic strains of *V. parahaemolyticus* can be considered ubiquitous in the marine environment.

The rapid emergence of *V. parahaemolyticus* non-O3:K6 serogroups carrying pandemic markers (Figure 1) indicates predisposition of *V. parahaemolyticus* to genetic change. Jones and colleagues recently reported isolating clinical isolates negative for *tdh*, *trh*, and T3SS2, indicating the *tdh* and/or *tdh* and T3SS2 genes are not necessarily predictive of pathogenic potential (Jones et al., 2012).

In summary, review of the literature shows a higher level of complexity in virulence potential for *V. parahaemolyticus* than previously known and draws attention to the value of reliable virulence markers. How can a strain be identified as pandemic or merely pathogenic? The search for pandemic strains might well prove to be very complicated if horizontal gene transfer is as extensive in *V. parahaemolyticus* as it is in *V. cholerae*. Strains representing pandemic, non-pandemic but pathogenic, and non-pathogenic strains of *V. parahaemolyticus* must be rigorously analyzed to assess the role of mobile genetic elements in *V. parahaemolyticus* virulence. This is especially important as horizontal transfer of mobile genetic elements may lead to emergence of new pandemic clones with expanded ecological persistence, infectivity, and dispersion.

## Author contributions

The project was conceived and designed by Daniela Ceccarelli, Nur A. Hasan, Anwar Huq, and Rita R. Colwell. The paper was written by Daniela Ceccarelli. All authors discussed, read, contributed to and approved the final manuscript.

### Conflict of interest statement

The authors declare that the research was conducted in the absence of any commercial or financial relationships that could be construed as a potential conflict of interest.
